# Neuronal oscillations and speech perception: critical-band temporal envelopes are the essence

**DOI:** 10.3389/fnhum.2012.00340

**Published:** 2013-01-04

**Authors:** Oded Ghitza, Anne-Lise Giraud, David Poeppel

**Affiliations:** ^1^Biomedical Engineering, Boston UniversityBoston, MA, USA; ^2^Department of Neuroscience, University Medical CentreGenève, Switzerland; ^3^Department of Psychology, New York UniversityNew York, NY, USA

**Keywords:** intelligibility, syllabic parsing, cascaded neuronal oscillations, hierarchical window structure, critical-band envelopes

## Abstract

A recent opinion article (Neural oscillations in speech: do not be enslaved by the envelope. Obleser et al., 2012) questions the validity of a class of speech perception models inspired by the possible role of neuronal oscillations in decoding speech (e.g., Ghitza, 2011; Giraud and Poeppel, 2012). The authors criticize, in particular, what they see as an over-emphasis of the role of temporal speech envelope information, and an over-emphasis of entrainment to the input rhythm while neglecting the role of top-down processes in modulating the entrainment of neuronal oscillations. Here we respond to these arguments, referring to the phenomenological model of Ghitza (2011), taken as a representative of the criticized approach.

There is a remarkable correspondence between the time scales of phonemic, syllabic, and phrasal (psycho)-linguistic units, on the one hand, and the periods of the gamma, beta, theta, and delta oscillations, on the other. This correspondence has inspired recent hypotheses on the potential role of neuronal oscillations in speech perception (e.g., Poeppel, 2003; Ahissar and Ahissar, 2005; Ghitza and Greenberg, 2009; Ghitza, 2011; Giraud and Poeppel, 2012; Peelle and Davis, 2012). In particular, in an attempt to account for counterintuitive behavioral findings on the intelligibility of time-compressed speech as a function of “repackaging” rate (Ghitza and Greenberg, 2009), a cortical computation principle was proposed according to which the speech decoding process is controlled by a time-varying, hierarchical window structure synchronized with the input (Ghitza, 2011). The window structure was assumed to be realized by a neuronal mechanism, with cascaded oscillations at the core, capable of tracking the input pseudo-rhythm embedded in the critical-band envelopes of the auditory stream. In the model, the theta oscillator is the “master” and the other oscillators entrain to theta. The key property that enabled an explanation of the behavioral data is the capability of the window structure to stay synchronized with the input; performance is high so long as the oscillators are phase-locked to the input rhythm (and within their intrinsic frequency range), and drops once the oscillators are out of their preferred temporal regime (e.g., exceed their boundaries). Giraud and Poeppel (2012) described a neurophysiological model which parallels Ghitza's phenomenological model, and discussed new neuroimaging evidence illustrating the operations and computations implicated in this oscillatory framework.

In a recent opinion article, Obleser et al. (2012) criticize the proposed model. Addressing Giraud and Poeppel (2012) they write: “… while we enjoy the ‘perspective’ Giraud and Poeppel (2012) are offering, it seems to oversimplify the available evidence …” in the following three respects: (1) lack of precision in defining the range of the neuronal oscillations and lack of specificity about the relationship between them (in particular, the boundaries between delta and theta or theta and alpha), hence the overlook of important functional differentiations between these oscillations, (2) over-emphasis of the role of temporal speech-envelope information in speech perception, and (3) over-emphasis of entrainment to the input pseudo-rhythm while neglecting the role of top-down processes in modulating the entrainment of neuronal oscillations.

It should be noted, at the outset, that we were aiming to offer a model for *some* critical computations in parsing and decoding speech, not a programmatic one-size-fits-all solution for *all* of speech comprehension. Nevertheless, Obleser et al. raise some important follow-up questions. For the sake of argument, items (1) and (3) can be grouped into one category, namely the potential implication of the omission of alpha-theta and delta-theta interactions on the validity of the cortical computation principle at the core of our model. In the following we briefly address these arguments by referring to the phenomenological model proposed by Ghitza (2011).

## The role of the temporal envelope: full-band vs. cochlear output

When discussing the possible role of the temporal envelope of speech for perception, the term “envelope” is often taken to refer to the envelope of the waveform itself, i.e., of the full-band signal. We argue, in concurrence with Obleser et al., that such practice is problematic, and that one should refer to the information at the cochlear output level (Ghitza, 2011, 2012)^1^. This is the case because, by necessity, the sole acoustic input available to the auditory brain is the information conveyed by the auditory nerve. What are the consequences of referring to the full-band signal, instead?

**Figure 1:**
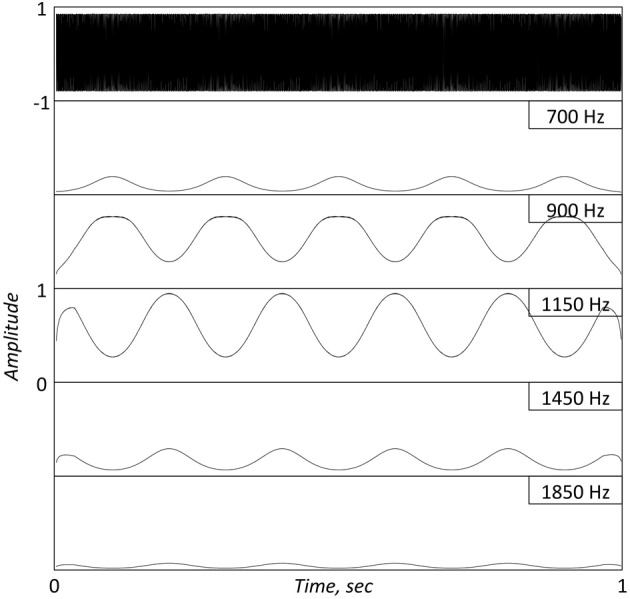
**Top panel.** A 1 s long FM stimulus with a 1 KHz carrier, modulated by a 5 Hz sinusoid. **Bottom Panels**: Simulated Inner Hair Cell (IHC) responses, low-pass filtered to 50 Hz, at five successive center frequencies (CFs) surrounding the carrier location. The cochlear filters are modeled as linear gammatone filters and the IHC as a half-wave rectifier followed by a low-pass filter, representing the reduction of synchrony with CF. Note the re-generation of the modulating signal at the cochlear output.

**Figure 2:**
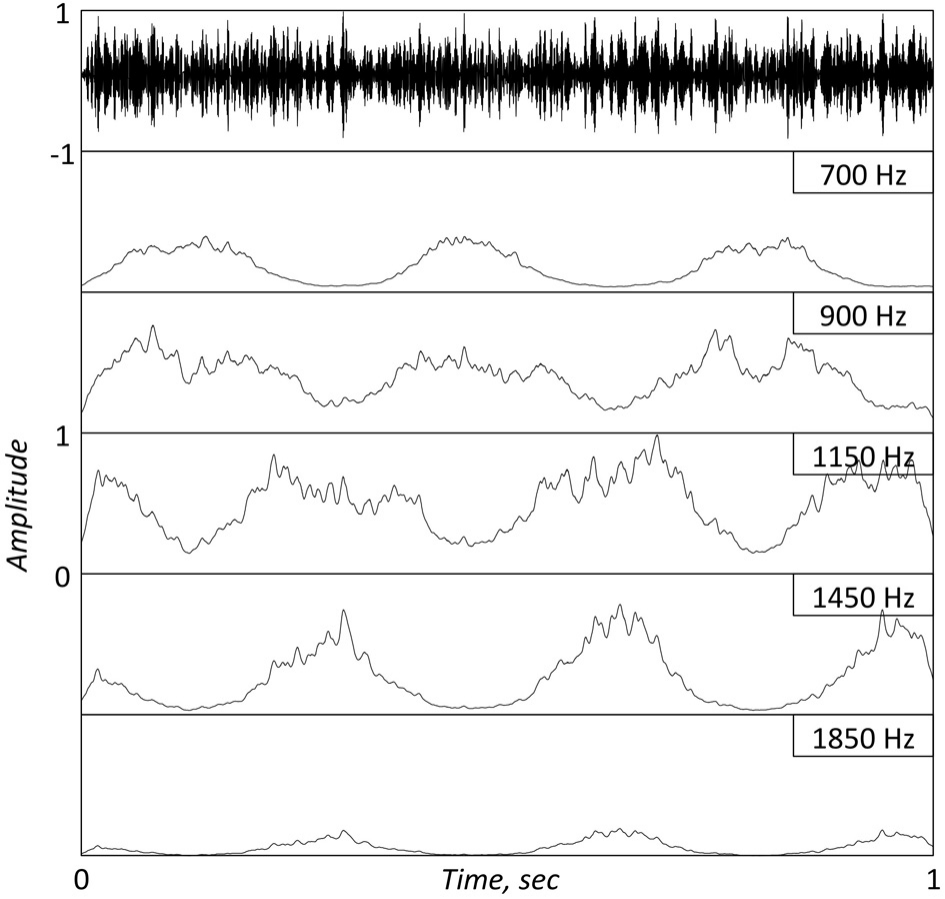
**Top panel.** A 1 s long FM stimulus with a complex carrier centered at 1 KHz, modulated by a 3 Hz sinusoid. [Provided by Obleser; see description at Obleser et al. (2012)]. **Bottom Panels**: Same as in bottom panels of Figure 1. Note the re-generation of the modulating signal at the cochlear output. The jitters in the IHC response are a reflection of the non-flat temporal envelope of the full-band stimulus.

Consider the argument raised by Obleser et al., embodied in their Figure 1 (and is the catalyst for the title: “… don't be enslaved by the envelope”). How come, they ask, are peaks observed at the frequency of the modulating signal in both the EEG phase coherence and the EEG power, even though the envelope of the FM stimulus (their Figure 1A) is flat^2^? A theorem in the field of communications provides an analytic answer to this question. The theorem determines that if a signal φ(*t*) is a band-limited signal, and if the FM signal *A*·cos[φ(*t*)] is the input to a band-pass filter with a bandwidth in the order of the bandwidth of φ(*t*), then the filter's output has an envelope that is related to φ(*t*) (e.g., Rice, 1973)^3^. A corollary to this theorem [noticed by Ghitza (2001)] is that if the band-pass filter represents a cochlear filter, then the envelope information at the cochlear output (i.e., the information available to the brain) is some non-flat, non-linear function of φ(*t*)! (This corollary was later validated psychoacoustically, e.g., Gilbert and Lorenzi, 2006.) In Obleser et al. three FM stimuli were used, with 500 Hz wide complex carrier signals centered on one of three frequencies (800, 1000, and 1200 Hz), and with a modulating signal of 3 Hz. Since critical bands at these frequencies are 100–150 Hz wide, such signals, when presented to the listener ear, will result in critical-band outputs with non-flat temporal envelopes that are related to the 3 Hz modulation signal^4^. Figures 1 and 2 illustrate this phenomenon using a FM stimulus with a 1 KHz carrier modulated by a 5 Hz sinusoid, and a stimulus provided by Obleser et al. (2012, Figure 1A), respectively.

Next, consider our current understanding of the relationship between a driving, full-band signal (fine structure and envelope) and the properties of the auditory nerve firing patterns it stimulates. This understanding is better, in particular, for auditory nerve fibers with high characteristic frequencies (CFs), where the synchrony of neural discharges to frequencies near the CF is greatly reduced due to the physiological limitations of the inner hair cells in following the carrier (i.e., fine structure) information. At these frequencies, temporal information is preserved by the instantaneous average rate of the neural firings, which is related to the temporal envelope of the underlying driving cochlear signal. Obviously, there is no distinct boundary between the low-CF and high-CF auditory nerve regions. Rather, the change in properties is gradual, and a reasonable assumption is that the region of transition is around 1200 Hz. Recalling that a significant amount of acoustic-phonetic information pertaining to intelligibility resides at the frequency range above 1200 Hz, the prominent speech information available to the brain are the temporal envelopes at the cochlear output.

## The role of alpha-theta and delta-theta interaction

Obleser et al. criticize the lack of precision in defining the range of the neuronal oscillations and the lack of specificity about the relationship between them. We acknowledge the inconsistencies in specifying the frequency range of the theta in the neurophysiological version of the model but note that it stems from inconsistencies inherent in the neurophysiological data. In the phenomenological version of the model (Ghitza, 2011) the oscillators in the array (theta, beta, and gamma) are related and cascaded (inspired by nesting, e.g., Schroeder and Lakatos, 2009), with the frequency of theta (the master oscillator) restricted to a range between 4 and 10 Hz, the frequency of beta to be a multiple (set to 4) of theta (16 and 40 Hz)^5^ and the frequency of gamma—a multiple of 4 of beta. For the purpose of demonstrating the crucial role played by the proposed cortical computation principle in speech perception such degree of specificity in defining the oscillatory array is sufficient, enough to account for the complex behavioral data of Ghitza and Greenberg^6^. Three points are noteworthy. First, as pointed out in Ghitza (2011), the beta-theta ratio and the gamma-beta ratio should be set up in accord with neurophysiological data. At present, there is a lack of a unanimous agreement on the frequency range of these oscillators; nevertheless, we believe that our choice is within reason. Second, in the model, the window structure comprises two timescales defined by the theta and the beta cycles. The role of gamma is different: it determines the time-instances at which the sensory information is sampled within the beta cycle [see Appendix in Ghitza (2011)]. Finally, we realize that no hypothesis (or a model) about internal physiological processes can be fully validated using only psychophysical measurements. Nevertheless, the capability of the model to explain the behavioral data establishes a behavioral context for future brain-imaging experiments using comparable speech material.

Obleser et al. argue against our strong focus on entrainment by the input syllabic rhythm; they suggest that “… in line with the mantra ‘correlation ≠ causation,’ it is also possible that phase-locking decreases are caused by poor intelligibility [the chicken and egg problem].” To illustrate this argument they cite two studies, by Obleser and Weisz (2012) and Peelle et al. (2012). It is interesting to examine these examples which, in our view, actually *reinforce* the basic assumptions of our model. In the first example, Obleser and Weisz measured alpha and theta MEG power in response to degraded speech, as a function of the amount of degradation. In the other, Peelle et al. measured coherence between theta, on the one hand, and the temporal envelope of the full-band speech stimuli, on the other, as a function of the amount of linguistic information in the stimuli. In both studies, stimuli were generated by a noise-excited channel vocoder (Shannon et al., 1995). This system enables the control of the amount of acoustic-phonetic information carried by the stimulus (achieved by changing the number of channels) while keeping the temporal envelope virtually unchanged. Indeed, an increase in negative correlation of the alpha and the theta power was observed with the increase of degradation (Obleser and Weisz, 2012), and an increase of the coherence between theta and the temporal envelope was observed with the increase of linguistic information (Peelle et al., 2012). Strikingly, in both studies a robust theta activity is registered even for the condition with the most severe degradation (i.e., absence of linguistic information). We conclude, therefore, that temporal envelope fluctuations alone, with negligible amount of acoustic-phonetic information, are sufficient to evoke theta activity of a considerable power (see also Howard and Poeppel, 2010), and that adding extra acoustic-phonetic information enhances the presence of theta, seemingly due to a delta-to-theta and alpha-to-theta feedback. It was suggested previously (Ghitza, 2011; Giraud and Poeppel, 2012) that the reasons for the assignment of the theta as the master oscillator are the strong presence of energy fluctuations in the range of 3–10 Hz in the speech acoustics (such strong presence is crucial for a robust tracking of the input rhythm by the cascaded array), and the psychophysical evidence on the importance to intelligibility of modulations in the range of 3–10 Hz (e.g., Houtgast and Steeneken, 1985; Ghitza, 2012). The findings by Obleser and Weisz (2012), and Peelle et al. (2012) provide further support for this view.

Finally, Obleser et al. further caution that the omission of the possibility that “… delta vs. theta bands, or theta vs. alpha bands, do subserve discontinuous, separable processing modes in the auditory and speech-processing domain … hinder rather than benefit our understanding.” Given the crucial role of the theta oscillations in our model (theta being the master) we concur with the importance of incorporating these intra-band interactions into the model. In our view the delta oscillation, in particular, plays an important role in *prosodic* parsing, which pertains to sequences of syllables and words hence tapping contextual effects^7^. As such, we believe that the delta oscillator interacts with the theta in a top-down fashion. Leaving aside the lack of knowledge on how a delta-theta interaction is carried out cortically, recall that our model is restricted to recognizing syllables in spoken sentences without context^8^. As for the possible role of the alpha oscillation, it may play a specific role in auditory gating (Sadaghiani et al., 2012), out of our scope.

### Conflict of interest statement

The authors declare that the research was conducted in the absence of any commercial or financial relationships that could be construed as a potential conflict of interest.

## Footnotes

^1^Obleser et al. caution against over-emphasizing the role of the temporal envelope of speech in speech perception and correctly point to the importance of the spectrotemporal modulations of the stimulus. Recalling that modulation spectra are derived from critical-band envelopes, we opted to explicitly reemphasize the crucial role of critical-band envelopes in speech perception.

^2^It is worth mentioning that a wealth of empirical data show that cortical responses entrain to FM sounds, from the delta range to the gamma range (e.g., Luo et al., 2006, 2007; Ding and Simon, 2009).

^3^The analytic expressions derived by Rice relate the output envelope and φ(*t*) in a complex, nonlinear manner.

^4^In this manuscript the terms “critical-band filter” and “cochlear filter” are synonymous. Strictly speaking, the critical band filters are auditory filters derived from psychophysical data rather than cochlear filters, derived from physiological measurements. In the context of our discussion, however, this difference is not relevant.

^5^It is noteworthy that oscillations in the high end of this range may also be considered low-gamma oscillations.

^6^The data—difficult to explain by conventional models of speech perception but emerging naturally from the architecture of our model—show that intelligibility of speech that is time-compressed by a factor of 3 (i.e., a high syllabic rate) is poor (above 50% word error rate), but is substantially restored when the information stream is re-packaged by the insertion of silent gaps in between successive compressed-signal intervals—a counterintuitive finding.

^7^Note that there are differences in delta responses across languages due to the different usage of stress. In English and German, for example, phrasal responses are emphasized but in French, syllabicity remains dominant.

^8^Ghitza (2011) aimed at developing a model capable of accounting for the data of Ghitza and Greenberg; they used speech material comprised of naturally spoken, semantically unpredictable sentences (i.e., sentences without context).
